# Alleviation of DSS-induced colitis in mice by a new-isolated *Lactobacillus acidophilus* C4

**DOI:** 10.3389/fmicb.2023.1137701

**Published:** 2023-04-20

**Authors:** Qianqian Liu, Wenwen Jian, Lu Wang, Shenglin Yang, Yutian Niu, ShuaiJing Xie, Kim Hayer, Kun Chen, Yi Zhang, Yanan Guo, Zeng Tu

**Affiliations:** ^1^Department of Pathogen Biology, College of Basic Medical Science, Chongqing Medical University, Chongqing, China; ^2^International Medical College, Chongqing Medical University, Chongqing, China; ^3^Leicester Medical School, University of Leicester, Leicester, United Kingdom; ^4^College of Foreign Languages, Chongqing Medical University, Chongqing, China

**Keywords:** *Lactobacillus acidophilus*, ulcerative colitis, probiotics, intestinal barriers, intestinal microbiota

## Abstract

**Introduction:**

Probiotic is adjuvant therapy for traditional drug treatment of ulcerative colitis (UC). In the present study, *Lactobacillus acidophilus* C4 with high acid and bile salt resistance has been isolated and screened, and the beneficial effect of *L. acidophilus* C4 on Dextran Sulfate Sodium (DSS)-induced colitis in mice has been evaluated. Our data showed that oral administration of *L. acidophilus* C4 remarkably alleviated colitis symptoms in mice and minimized colon tissue damage.

**Methods:**

To elucidate the underlying mechanism, we have investigated the levels of inflammatory cytokines and intestinal tight junction (TJ) related proteins (occludin and ZO-1) in colon tissue, as well as the intestinal microbiota and short-chain fatty acids (SCFAs) in feces.

**Results:**

Compared to the DSS group, the inflammatory cytokines IL-1β, IL-6, and TNF-α in *L. acidophilus* C4 group were reduced, while the antioxidant enzymes superoxide dismutase (SOD), glutathione (GSH), and catalase (CAT) were found to be elevated. In addition, proteins linked to TJ were elevated after *L. acidophilus* C4 intervention. Further study revealed that *L. acidophilus* C4 reversed the decrease in intestinal microbiota diversity caused by colitis and promoted the levels of SCFAs.

**Discussion:**

This study demonstrate that *L. acidophilus* C4 effectively alleviated DSS-induced colitis in mice by repairing the mucosal barrier and maintaining the intestinal microecological balance. *L. acidophilus* C4 could be of great potential for colitis therapy.

## 1. Introduction

Inflammatory bowel disease (IBD) is a group of chronic inflammatory disease of the gastrointestinal tract, including ulcerative colitis (UC) and Crohn’s disease (CD). UC is persistent and confined to the colon, whereas CD lesions are intermittent and involve all parts of the digestive tract (Silverberg et al., 2005; Baumgart and Carding, 2007). The clinical manifestations of UC are diarrhea, abdominal pain, and severe bloody stools (Silverberg et al., 2005). Similar symptoms can be induced in mice with the addition of dextran sulfate sodium (DSS), a routine that has been used in UC researches currently. Although the etiology of UC remains unknown, damage to the mucosal barrier, imbalance of the pro-inflammatory and anti-inflammatory responses, and intestinal microbiota disorders are considered risk factors (Izcue et al., 2009; Turner, 2009; McCauley and Guasch, 2015; Xiao et al., 2016).

Aminosalicylic acid, hormones, and immunosuppressive agents are commonly used in UC treatment (Regueiro et al., 2006; Khan et al., 2011; Ford et al., 2012). However, the therapeutic efficacy is not satisfactory and patients suffer from adverse drug reactions. Hence, the treatment with probiotics alone and the combination of probiotics with traditional drugs for UC have attracted great attention (Lee et al., 2015; Jeong et al., 2019). One study showed that the clinical relief of UC patients with *Lactobacillus rhamnosus* GG alone or in combination with 5-Aminosalicylic acid (5-ASA) was significantly longer compared with the anti-inflammatory drug 5-ASA group alone (Saez-Lara et al., 2015). *Lactobacillus* and *bifidobacteria* administration as adjuvant therapy also significantly improved the disease course of UC patients and relieved of clinical symptoms (Saez-Lara et al., 2015; Basso et al., 2018). When treating UC patients with probiotics and 5-ASA, both were found to show the same effect in clinical, endoscopic, and histological aspects (Kruis et al., 2004, 1997). In addition, an analysis of nine trials showed no significant effect of probiotics on CD, while another analysis of 18 trials showed a significant effect of probiotics in patients with UC under different conditions (Ganji-Arjenaki and Rafieian-Kopaei, 2018), which suggests that probiotics are more effective in treating UC than CD. Treatments using probiotics tend to be more effective in UC rather than CD patients, and the immunity state of patients, and intestinal microbiota could define the difference of therapeutic effects (Jakubczyk et al., 2020). *E. coli Nissle* 1917 could relieve UC, according to the European Crohn’s and Colitis Organization guidelines (Travis et al., 2008). A probiotic mixture including eight live bacterial strains, VSL#3, could regulate the intestinal barrier function, induce and maintain remission of patients with mild to moderate UC (Miele et al., 2009; Basso et al., 2018; McIlroy et al., 2018). *L. reuteri* ATCC 55730 enemas improved the symptoms in children with mild to moderate UC. In addition, many studies showed probiotics might modulate gut microbiota composition by inhibiting the growth of pathogenic gut microorganisms (Shiba et al., 2003; Sartor, 2004; Servin, 2004; Constante et al., 2017). Probiotics could produce short-chain fatty acids (SCFAs) that are essential to maintain the integrity of intestinal mucosa and have the potential anti-inflammatory and anti-cancer functions (Ewaschuk et al., 2006; Kuhbacher et al., 2006; Collado et al., 2007; Tannock, 2010; Ghouri et al., 2014; Constante et al., 2017; Basso et al., 2018; Khan et al., 2019). Therefore, probiotics could be a probable supplement for UC treatment with a comprehensive mechanism of action.

*Lactobacillus acidophilus* generally found in the gastrointestinal tract and mouth of human and animal (Liévin-Le Moal and Servin, 2014) is often added to yogurt, milk, and milk powder formulations as a food supplement because of its probiotic properties (Altermann et al., 2005). The activity of CD4-expressing T cells and type 3 innate lymphoid cells (ILC 3) is enhanced by supplementing *L. acidophilus* BIO5768, which plays a crucial role in the barrier function and tissue repair (Lindemans et al., 2015; Kumar et al., 2016). Probiotics from different sources have considerable resistance to acid and bile salt, and their beneficial functions are distinct for colitis treatment. Therefore, it is imperative and meaningful to investigate new isolates with good performance for DSS-induced colitis treatment.

In this study, a new strain *L. acidophilus* C4 was isolated and selected for being resistant analysis of acid and bile salt. *L. acidophilus* C4 was administered for 7 days to healthy mice without causing intestinal inflammation or epithelial barrier damage. The effect of the *L. acidophilus* C4 on DSS-induced colitis has been evaluated, and the underlying effects have been investigated.

## 2. Materials and methods

### 2.1. Materials and reagents

#### 2.1.1. Source of *L. acidophilus* C4

A total of 22 Lactobacillus were isolated from eight human’s stool samples and three sauerkraut water samples. And a well-performing strain of *L. acidophilus* C4 was finally screened and identified by acid and bile salt tolerance assay. The classification of the *L. acidophilus* C4 was determined by analysis of 16S rRNA sequence. The strain was then stored at China Center for Type Culture Collection (CCTCC) under conservation number CCTCC M 20211622. The PCR (Polymerase Chain Reaction) primers 27F and 1492R (Zhang et al., 2010) used for amplification of 16S rRNA are listed in Table 1 and the 16S rRNA sequence of *L. acidophilus* C4 (Supplementary Table 3) was uploaded to NCBI with the sequence number ON171206.

**TABLE 1:** Sequences of primers for 16S rRNA amplification.

	Forward (5′→3′)	Reverse (5′→3′)
27F/1492R	AGAGTTTGATCCTGGCTCAG	CTACGGCTACCTTGTTACGA
338F/806R	ACTCCTACGGGAGGCAGCAG	GGACTACHVGGGTWTCTAAT

Primers 27F/1492R to amplify 16S rRNA gene for *L. acidophilus* C4 and primers 338F/806R to amplify the 16S rRNA V3–V4 hypervariable region for microbiota dynamic evaluation.

#### 2.1.2. Determination of the intestinal colonization ability of *L. acidophilus* C4

The *L. acidophilus* C4 was inoculated in de Man, Rogosa, and Sharpe (MRS) broth, (Qingdao Hope bio Biotechnology Co., Ltd., Qingdao, China) MRS (pH 2) broth and MRS broth containing 0.3% bile salt, cultured at 37°C for 14 h. Then, the number of the bacterial colony was counted by plate counting to calculate acid and bile salt tolerance (Manini et al., 2016). The surface hydrophobicity of *L. acidophilus* C4 was detected by bacterial adhesion to hydrocarbons (BATH), and the self-cohesion detection method was done according to Angmo’s method (Angmo et al., 2016; Yang et al., 2020).

#### 2.1.3. Preparation of the bacterial suspension

The *L. acidophilus* C4 pure colony was inoculated with 5 ml MRS broth. After being anaerobic cultured at 37°C for 14 h, 1 ml of the bacteria-containing culture medium was taken and centrifuged at 4,000 *g* for 5 min before discarding the supernatant. The pellet was washed three times with phosphate-balanced solution (PBS), centrifuged at 4,000 *g* for 5 min. Finally, the pellet was resuspended in PBS to prepare a bacterial suspension.

### 2.2. Animal experimental methods

#### 2.2.1. Group design of the animal experiments

Twenty-four C57BL/6 mice (SPF grade, male, 5 weeks old) were purchased from Chongqing Medical University and were acclimatized to a constant temperature and humidity environment for 1 week. Twenty-four mice were randomly divided into four groups (6 mice/group): control (freely drinking beverage), DSS (DSS and freely drinking water), *L. acidophilus* C4 (DSS and 1*10^9^ CFU/ml C4 gavage beverage), and 5-ASA (DSS and 75 mg/kg 5-ASA gavage beverage). 5-ASA (Shanghai Aifa Pharmaceutical Co., Ltd., Shanghai, China) is a regularly used medicine in the clinical treatment of UC and was utilized as a positive control in this study. 2.5% of DSS (w/v) (MW = 36,000–50,000 Da, See bio Biotech Co., Ltd., Shanghai, China) was added to the drinking water of the mice, and each group except the control group was given 2.5% DSS water for seven consecutive days, and the water was changed every 2 days. After 7 days, the mice acute colitis model was established. During the period of building the colitis model, *L. acidophilus* C4 and 5-ASA were orally added once a day for 7 days to C4 group and 5-ASA group, respectively. Mice were weighed daily during the dosing time, and the disease activity index (DAI) was determined (see below). On the seventh day, fresh water replaced the DSS for 24 h. After feeding, the mice were sacrificed, and the following experiment was performed. All experimental processes were approved by the Ethics Committee of Animal Experiments of Chongqing Medical University (No. 2022178).

#### 2.2.2. DAI score of mice

The DAI score was calculated according to the established scoring criteria of Murthy et al. (1993). There are three components in the DAI: weight change, blood in the stool, and stool character. The DAI was calculated as follows:


DAI⁢value=

{(thescoreofweightloss)+(thescoreofgrossbleeding)

+(thescoreofstoolconsistency)}/3

Scoring criteria were as follows: body weight loss (0: none, 1: 1–5, 2: 5–10, 3: 10–15, 4: >15%); stool consistency (0: normal, 1: loose stool, 2: loose stool, 3: diarrhea, 4: diarrhea); blood in the stool (0: occult blood test negative, 1: occult blood test negative, 2: weak positive detection of occult blood, 3: occult blood test strong positive, 4: gross bleeding).

#### 2.2.3. Macroscopic observation of colon

The colon length was measured after the mice were sacrificed. Then, 0.5 cm of colon tissue was taken 1 cm away from the end of the colon and immersed in 4% paraformaldehyde for 24 h. After more than 24 h, the wax-soaked tissues were dehydrated, embedded, sectioned, and stained with hematoxylin and eosin (H&E). Finally, the slices were sealed and examined by microscopy, and images were collected and analyzed.

#### 2.2.4. Determination of tissue cytokines and antioxidant enzymes

Total RNA was extracted from colon tissue (tissue material was taken for 0.5–1.2 cm from the end colon) using Trizol reagent (Invitrogen By Life Technology Inc., CA, USA) following the manufacturer’s instructions. Reverse transcription of RNA into cDNA was performed according to the manufacturer’s instructions (Takara Biomedical Technology Co., Ltd., Nojihigashi, Japan). The relative expression of cytokine mRNAs (TNF-α, IL-1β, IL-6, Occludin, and ZO-1) was detected by quantitative real-time polymerase chain reaction (qRT-PCR). The levels of nitric oxide (NO), malondialdehyde (MDA), glutathione (GSH), and superoxide dismutase (SOD) in colon homogenate (tissue material was taken for 1.3–2.5 cm from the caudal colon) were detected using Beyotime (Beyotime Biotechnology Co., Ltd., Shanghai, China) kits, and catalase (CAT) was measured by the product of Beijing Solarbio Science and Technology Co., Ltd (Beijing, China). The specific qRT-qPCR primers for target genes are shown in Table 2 (Hu et al., 2020).

**TABLE 2:** Sequences of primers for qRT-PCR.

	Forward (5′→3′)	Reverse (5′→3′)
IL-1β	GAAATGCCACCTTTTGACAGT	TGGATGCTCTCATCAGGACA
IL-6	TCCAGTTGCCTTCTTGGGAC	AGACAGGTCTGTTGGGAGTG
TNF-α	CAGGCGGTGCCTATGTCTC	CGATCACCCCGAAGTTCAGT
Occludin	CACACTTGCTTGGGACAGAG	TAGCCATAGCCTCCATAGCC
ZO-1	CTTCTCTTGCTGGCCCTAAAC	TGGCTTCACTTGAGGTTTCTG

#### 2.2.5. Western blot analysis

Tissues’ total protein (tissue material was taken for 2.6–3.2 cm from the caudal colon) was extracted by homogenizing them with lysis buffer. Protein concentrations were determined by bicinchoninic acid (BCA) protein analysis kits (Beyotime Biotechnology Co., Ltd., Shanghai, China).

A total of 30 μg proteins were subjected to 6–12% sodium dodecyl sulfate-polyacrylamide gels (SDS-PAGE) electrophoresis and then transferred to 0.45 μm polyvinylidene fluoride (PVDF) membranes (Beyotime Biotechnology Co., Ltd., Shanghai, China), The membranes were washed three times in TBST followed by blocking with 5% skim milk for 2 h at room temperature. Then, the membranes were incubated with primary antibodies overnight at 4°C. Primary antibodies and their dilution concentrations were as follows: occludin (1:1000, Beyotime Biotechnology Co., Ltd., Shanghai, China), ZO-1 (1:500, Affinity Biosciences Inc., OH, USA), and GAPDH (1:2000, Proteintech Group Inc., Rosemont, USA). Subsequently, the membranes were washed with TBST three times and incubated with horseradish peroxidase-conjugated (HRP-conjugated) secondary antibodies (diluted 1:5000) for 1 h at room temperature. Finally, the immune complexes were visualized on Amersham Imager 600 chemiluminescence instrument (GE Healthcare Bio-Sciences AB, Uppsala, Sweden) by an enhanced chemiluminescence kit (Beyotime Biotechnology Co., Ltd., Shanghai, China).

#### 2.2.6. Determination of SCFAs levels in faeces

A total of 25 mg solid sample was added to 500 μl water containing 0.5% phosphoric acid, and then frozen and grounded at 50 Hz twice for 3 min, followed by sonicating for 10 min and centrifuged at 13,000 *g* for 15 min at 4°C. 400 μl of the supernatant was aspirated and transferred to a new tube, which already had 0.2 ml *N*-butanol solvent containing internal standard 2-ethylbutyric acid (10 μg/ml) for exaction. The supernatant was vortexed for 10 sec, treated with ultrasound at 4°C for 10 min, followed by centrifugation at 13,000 *g* for 5 min at 4°C, and then were carefully transferred to a new tube for further detection. Detection was conducted using an Agilent 8890B-7000D GC mass spectrometer (Agilent Technologies Inc., Santa Clara, CA, USA) and an HP FFAP capillary column (Agilent J&W Scientific, Folsom, CA, USA).

#### 2.2.7 Analysis of intestinal microbiota

The unwashed intestinal contents of the mice were collected and placed in a 1.5 ml of sterile tube, snap frozen in liquid nitrogen, and stored at −80°C for further use. Total fecal DNA was extracted for concentration and purity detection, and DNA extraction quality was detected by 2% agarose gel electrophoresis. The V3-V4 hypervariable region of the 16S rRNA gene was amplified by PCR primers 338F and 806R (Table 1; Liu et al., 2016) and then sequenced on the Illumina NovaSeq PE250 platform (Illumina, San Diego, CA, USA) according to the standard protocols by Majorbio Bio-Pharm Technology Co., Ltd. (Shanghai, China). Quality control and splicing of raw sequences were performed by fastp 0.19.6 (Chen et al., 2018)^1^ and FLASH 1.2.11 (Magoc and Salzberg, 2011)^2^ software, respectively. OTU (Operational taxonomic unit) clustering of sequences with 97% similarity and elimination of chimeras was performed by UPARSE (Stackebrandt and Goebel, 1994; Edgar, 2013) software. Then, bioinformatics analysis was performed on the cloud platform of Majorbio Bio-Pharm Technology Co., Ltd., Shanghai, China.

### 2.3. Statistical analysis

Statistical analysis of the data in the text was performed on SPSS software 26.0. The data are presented as mean ± standard deviation. For testing differences between groups, data conforming to a normal distribution were analyzed by one-way ANOVA; otherwise, data were assessed using the rank sum test followed by Bonferroni’s *post hoc* analysis. SCFAs data were log-transformed before testing, and correlation heat maps were analyzed based on Spearman’s algorithm. The graph plotting was performed using GraphPad Prism 9.0. *P* < 0.05 was considered to be statistically significant.

## 3. Results

### 3.1. *L. acidophilus* C4 survives and colonizes the intestine

We explored the viability of *L. acidophilus* C4 in the circumstances of high bile salts and strong acids. A survival rate of 53.57% was observed in the MRS broth (pH 2) and 80% in the MRS broth containing 0.3% bile salt. These results support the colonization of the *L. acidophilus* C4 into the gut environment and survival. In addition, surface hydrophobicity and self-cohesion can reflect the adhesive ability of bacteria. The surface hydrophobicity and self-cohesion of *L. acidophilus* C4 are 92.08 and 84.94%, respectively, indicating a good capacity of *L. acidophilus* C4 adhesion onto the intestine (Supplementary Table 1).

### 3.2. *L. acidophilus* C4 alleviates weight loss and reduces DAI scores

**FIGURE 1:**
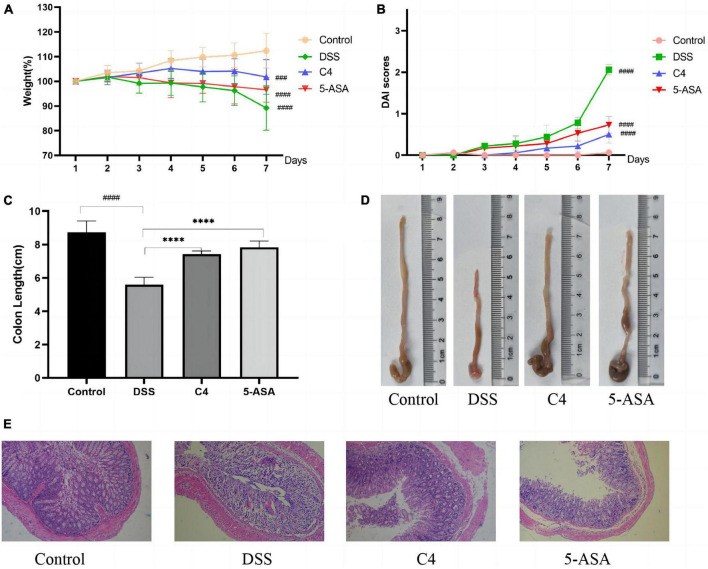
Effect of the *L. acidophilus* C4 on the colon and weight in DSS-induced colitis mice model. Data are subjected to one-way ANOVA statistics and presented as mean ± standard deviation (*n* = 6). **(A)** Changes in body weight. **(B)** Disease activity index scores. **(C,D)** Image of colon length. **(E)** Representative micrographs of H&E staining of colon tissue from the four groups of mice (400 fold magnification). ****P < 0.0001 vs. DSS group; ^###^*P* < 0.001 and ^####^*P* < 0.0001 vs. Normal group.

To investigate the effect of *L. acidophilus* C4 on body weight changes and DAI scores in DSS-induced colitis mice, we recorded the body weight and DAI scores (Figure 1). The body weight of the mice in the DSS group started to decrease on day three (Figure 1A). On the contrary, the weight of the C4 group appeared increasing from day one to day four, and the weight gently decreased after day five. The DAI scores (Figure 1B) showed the severity of colitis, including the degree of weight loss, the presence of blood in the stool, and the characteristics of the stool. The DAI value of the DSS group started to rise and surpass that of the control group significantly (*P* < 0.05) on day three. In addition, the C4 group’s DAI value started to increase gradually, but the trend was significantly slower than the DSS group (*P* < 0.05). The findings suggest that *L. acidophilus* C4 can lessen diarrhea and weight loss in colitis mice induced by DSS.

### 3.3. *L. acidophilus* C4 improves the colon damage

To explore whether *L. acidophilus* C4 could reduce colon tissue damage, we measured the colon length of mice and stained the tissue. The result demonstrated that the colon length of the DSS group (5.6 ± 0.44 cm) was substantially shorter than that of the control group (8.73 ± 0.68 cm) (*P* < 0.05) in Figure 1C. The colon length of the C4 group (7.43 ± 0.18 cm) was significantly longer than the DSS group (*P* < 0.05), indicating that the *L. acidophilus* C4 strain may reduce colon shortening in DSS-induced colitis mice.

The mice in the control group exhibited intact colonic epithelium and a neat crypt structure, as observed by H&E colon staining (Figure 1E). Colon tissue in the DSS group displayed a significant number of interstitial inflammatory cells between the basal layer and the mucosal muscle, a defect in the villi, the loss of crypts, and the loss of mucous (Figure 1E). In contrast, the colon of mice treated with *L. acidophilus* C4 (Figure 1E) showed minor tissue damage, a typical crypt structure, a slight infiltration of inflammatory cells in the basal layer, and a largely intact mucosa. From these results, we can postulate that *L. acidophilus* C4 could mitigate DSS-induced intestinal tissue damage.

### 3.4. *L. acidophilus* C4 reduces the oxidative stress

**FIGURE 2:**
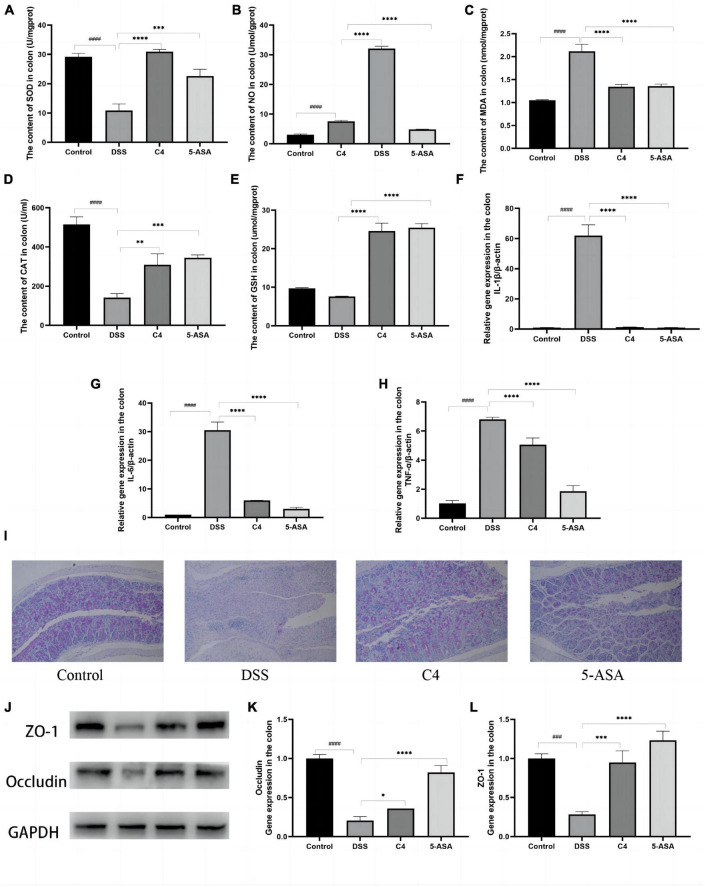
Effect of the *L. acidophilus* C4 on oxidative stress and pro-inflammation expression. Data are subjected to one-way ANOVA statistics and presented as mean ± standard deviation (*n* = 6). **(A–E)** Colon tissue oxidative stress. **(F–H)** Colon tissue inflammatory cytokines. **(I)** PAS staining of colonic tissue (400 fold magnification). **(J)** Protein expression of the TJ proteins Occludin and ZO-1 by Western blots. **(K,L)** mRNA levels of ZO-1 and Occludin by qRT-PCR. *P < 0.05, **P < 0.01, ***P < 0.001 and ****P < 0.0001 vs. DSS group; ^###^*P* < 0.001 and ^####^*P* < 0.0001 vs. Normal group.

Since the level of oxidative stress can reflect tissue damage, this study looked for evidence of intestinal tissue damage by measuring the level of oxidative stress. DSS treatment significantly increased MDA and NO levels (Figures 2B, C) compared to the control group (*P* < 0.05), but *L. acidophilus* C4 treatment substantially reduced these levels. SOD (*P* < 0.05), CAT (*P* < 0.05), and GSH were all considerably lowered in the DSS group. However, those levels (*P* < 0.05) were increased markedly, and SOD (*P* < 0.05) altered dramatically most after *L. acidophilus* C4 intervention (Figures 2A, D, E). These findings indicated that the anti-inflammatory impact of *L. acidophilus* C4 on DSS-induced colitis may be achieved by boosting antioxidant enzymes levels (SOD, CAT, and GSH) and decreasing oxidative stress (MDA and NO).

### 3.5. *L. acidophilus* C4 decreases inflammatory cytokines production in colon

Levels of pro-inflammatory cytokines in colon tissue were correlated with the severity of tissue inflammation. The levels of proinflammatory cytokines IL-1β, TNF-α, and IL-6 in the DSS group were much higher than that in the control group (*P* < 0.05). In contrast, those pro-inflammatory cytokines levels were significantly decreased in the C4 group (*P* < 0.05) compared to the DSS group (Figures 2F–H). These results pointed out that *L. acidophilus* C4 can lessen DSS-induced colitis by regulating the levels of inflammatory cytokines.

### 3.6. *L. acidophilus* C4 repairs the intestinal mucosal barrier

Mucin has a lubricating and saturating effect, which is required to form the mucosal barrier. PAS staining was used to ascertain the quantity and distribution of cup mucin. Figure 2I showed that mucin levels of the DSS group were considerably lower compared to the control group. In addition, the mucin content was increased after 5-ASA and *L. acidophilus* C4 treatment (Figure 2I). Occludin and ZO-1 are essential proteins that compose the tight junction (TJ) between colonic mucosal cells. The Expression of occludin and ZO-1 considerably dropped (*P* < 0.05) in the DSS group (Figures 2J–L), but *L. acidophilus* C4 feeding enhanced the expression of occludin and ZO-1. These findings indicated that *L. acidophilus* C4 could repair and protect the intestinal mucosal barrier by adjusting the expression of the mucosal barrier-related proteins occludin and ZO-1.

### 3.7. *L. acidophilus* C4 modulates the intestinal microbiota in the colon

**FIGURE 3:**
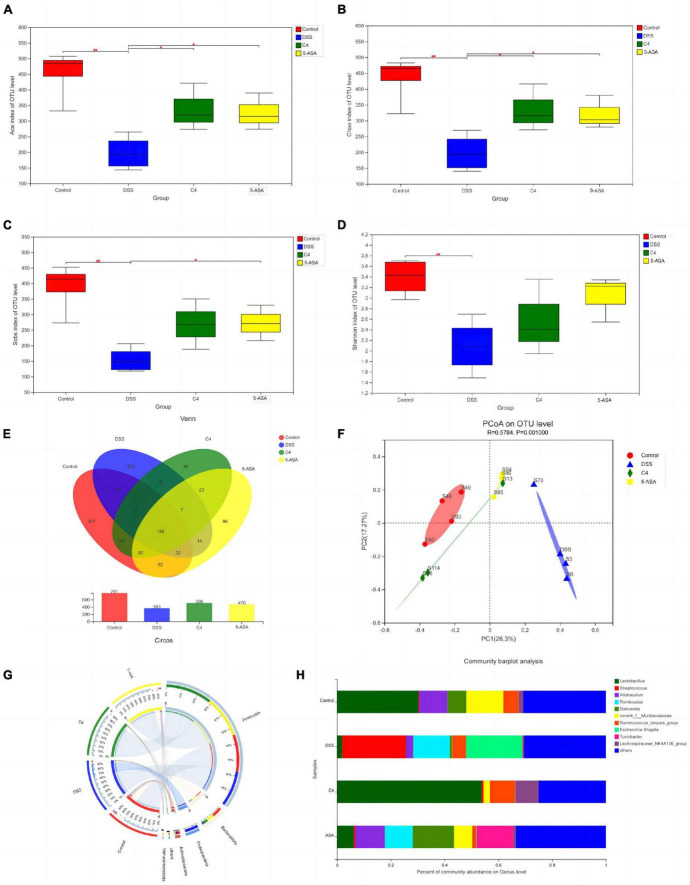
Effect of the *L. acidophilus* C4 on the gut microbial environment. Data are subjected to one-way ANOVA statistics and presented as mean ± standard deviation (*n* = 4). Three replicates were set for each group. **(A–D)** Alpha diversity of intestinal microbiota at the OTU level. **(E)** Venn diagram representing species composition analysis. **(F)** Beta diversity of intestinal microbiota. **(G)** Classification of intestinal microbiota composition at the phylum level. **(H)** Genus level gut microbiota bar graph analysis. *P < 0.05 vs. DSS group; ^##^*P* < 0.01 vs. Normal group.

**FIGURE 4:**
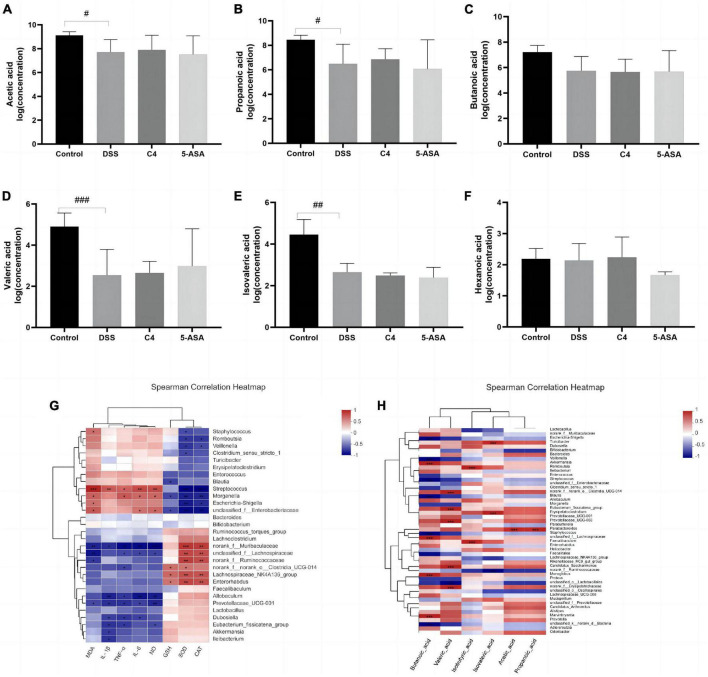
Effect of The *L. acidophilus* C4 on the production of SCFAs. Data are subjected to one-way ANOVA statistics and presented as mean ± standard deviation (*n* = 4). Three replicates were set for each group. **(A–F)** Changes in SCFAs levels in rat feces. **(G)** Heatmap of the correlation between inflammatory cytokines and oxidative stress on gut microbiota at gene level. **(H)** Heat map of the correlation between SCFAs and intestinal microbiota at gene level. ^#^*P* < 0.05, ^##^*P* < 0.01 and ^###^*P* < 0.01 vs. Normal group.

To assess the beneficial effects of *L. acidophilus* C4 on modifying the intestinal microbiota structure, the intestinal microbiota composition was analyzed using 16S rRNA gene sequencing. Alpha-diversity reflects the abundance and homogeneity in the community. The Chao, ACE, and Sobs (Supplementary Figure 1) indices refer to the number of bacterial species in a community regardless of the abundance of each species. Shannon indices are influenced by species richness and evenness in the sample and thus can reflect species homogeneity of the bacterial community in the sample (Rogers et al., 2016). In Figure 3, the Chao and Shannon indices in the DSS group were significantly lower than those in the control group. Intriguingly, those indices increased greatly in the C4 group (Figures 3A, B). The result suggested that *L. acidophilus* C4 can increase bacterial diversity and abundance. Subsequently, the microbiota composition in the four groups by quantifying the number of OTUs was investigated (Supplementary Table 2). The numbers of OTUs (Figure 3C) in control, DSS, C4, and 5-ASA groups were 781, 363, 506, and 470, respectively. We performed the principal coordinate analysis (PCoA) of OTU levels to evaluate differences in gut microbiota compositions between groups. In Figure 3D, the gut microbiota composition of C4 group was similar to that of the control group. In all four groups, the dominant bacteria belonged to the Firmicutes phylum, as illustrated in Figure 3E. The relative abundance of *Lactobacillus* and *Allobaculum* decreased in the DSS group, while *Streptococcus* and *Ruminococcus* increased (Figure 3F). The relative abundance of *Lactobacillus* and *Muribaculaceae* increased, whereas *Streptococcus* and *Ruminococcus* decreased (*P* < 0.05) in the C4 group (Figure 3G). The results indicated that *L. acidophilus* C4 increased the count of *Lactobacillus*. The spearman method examined the relationship between inflammatory variables and intestinal microbiota (Figure 4G). At the genus level, GSH, SOD (*P* < 0.05), and CAT levels were highly correlated with *Lachnospiraceae*, *Muribaculaceae*, and *Ruminococcaceae*, respectively. *Streptococcus* and *Morganella* were positively associated with MDA, IL-1β, IL-6, TNF-α, MDA, and NO levels.

### 3.8. *L. acidophilus* C4 increases the production of SCFAs

Short-chain fatty acids, crucial intestinal microbiota metabolites, have been proven effective in the treatment and prevention of colitis (Liu et al., 2021). Therefore, this study examined the concentration of the six SCFAs. Acetic acid, propionic acid, propionate acid, butyric acid, valeric acid, and caproic acid (Figures 4A–F). The contents of acetic acid, propionic acid, butyric acid and valeric acid were decreased in the DSS group compared to the control group. However, *L. acidophilus* C4 intervention was able to partially reverse the reduction of these SCFAs caused by DSS. The content of formic acid and acetate is associated with the abundance of *Wilhelm Ackermann* (Figure 4H). *Rumen* bacteria can affect propionic acid levels. These results suggested *L. acidophilus* C4 could promote the production of SCFAs and relieve the symptoms of intestinal inflammation.

## 4. Discussion

In the present study, we have demonstrated that a newly isolated *L. acidophilus* C4 in our lab not only can stably survive and colonize the intestinal environment but also has a great alleviation effect on the colitis symptoms in the DSS-induced mice model. The effect includes improvement of the intestinal pathology in mouse, reduction of tissue-related inflammatory cytokines and oxidative stress, and regulation of the intestinal barrier function. Those results revealed *L. acidophilus* C4 is potential probiotic for DSS-induced colitis treatment.

UC is becoming more and more pervasive throughout the world. The pathological factors of UC are the loss of the mucosal barrier and the imbalance of gut microbiota, which stimulates the gastrointestinal immune response (Porter et al., 2020). The intestinal mucosal barrier protects against bacterial invasion and maintains the immune function homeostasis of the gut (Kmiec et al., 2017). Pro-inflammatory cytokines, antigens, and pathogens disrupt the barrier (Nusrat et al., 2000; Capaldo and Nusrat, 2009), and pro-inflammatory cytokines IL-1β and IL-6 can amplify inflammation in UC (Zhou et al., 2019). Furthermore, the TJ structures play an essential role in stabilizing the intestinal mucosal barrier, which could close gaps between cells and prevent some harmful substances from entering the submucosa (Otani and Furuse, 2020). IL-1β could increase the permeability of intestinal TJs by decreasing occludin and cytoskeletal rearrangement (Suzuki et al., 2011). ZO-1 (Suzuki, 2020) and Occludin (Rao, 2009; Al-Sadi et al., 2011) are essential proteins constituting TJ structures and could indirectly reflect the integrity of the mucosal barrier.

Probiotics play an important role in the treatment of UC and were devoted to restoring the mucosal barrier-related proteins and protecting the intestinal barrier against the effect of inflammatory cytokines and infections. Our study showed that the newly isolated C4 strain could effectively reduce the expression levels of IL-1β, IL-6, and TNF-α, and upregulate the expression levels of ZO-1 and occludin to alleviate the symptoms in DSS-induced colitis mice. Previous studies have reported the relief of intestinal inflammation by beneficial bacteria. For example, *L. rhamnosus* CY12 strain alleviated the oxidative stress and the injured the intestinal TJ barrier function and inflammatory response in LPS-induced Caco-2 cells by regulating the levels of antioxidant enzymes (CAT, SOD, and GSH-Px) and expression of TJ proteins (Claudin, Occludin, and ZO-1) (Kim et al., 2018; Wu et al., 2018; Chen et al., 2019). Another probiotic VSL#3 could reduce hyperpermeability and inflammation of the colon (Mennigen et al., 2009) and increased the expression of TJ proteins (Dai et al., 2012). In addition, in an animal model of DSS-induced colitis, intake of live or heat-inactivated *rhamnose* OLL2838 ameliorates intestinal inflammation (Miyauchi et al., 2009). Those studies, together with our results, predispose the potential application of beneficial bacteria in UC therapy.

The innate immunity of the intestinal mucosa is significantly influenced by gut microbiota balance and the accompanying metabolic production. Interestingly, probiotics could enhance the SCFAs content by modulating intestinal microbiota, thereby strengthening the intestinal mucosal barrier. In our study, we explore the efficacy of *L. acidophilus* C4 administration in improving symptoms of DSS-induced colitis. Results showed the intestinal microbiota composition was altered distinctly, and then the production of SCFAs was further regulated. This effect of colitis alleviation is not unique. For instance, the structure of the polysaccharides could affect the microbiota composition (El et al., 2013), and gut microorganisms could catabolize polysaccharides to create SCFAs (Ge et al., 2021). Intestinal probiotics colonize on intestinal mucosa to form a bacterial barrier, which could effectively prevent harmful bacteria from entering intestinal mucosa and causing tissue damage (Salminen et al., 1998; Sanchez et al., 2014). Moreover, modulating the composition of the intestinal microbiota boosts the development of AMPs, TJs, and MUCs, regulates the release of cytokines, and limits the synthesis of LPS (Hiippala et al., 2018). SCFAs and other metabolites produced by the gut microbiota could protect the intestinal barrier (Cheng et al., 2022). The mucophilic bacterium *Akkermansia* is a good example to protect the intestinal barrier by acetate and propionate production (Hiippala et al., 2018). Unlike the results of other studies where strains alleviated DSS-induced colitis by a single effect (Chen et al., 2015; Huang et al., 2021), we have demonstrated that the protective effect of *L. acidophilus* C4 for the repair of the intestinal mucous is achieved by relieving colon tissue damage, reducing the level of tissue inflammatory factors, and regulating the oxidative stress in mice. In addition, *L. acidophilus* C4 can maintain the homeostasis of the intestinal microenvironment by regulating the composition and abundance of the intestinal microbiota and the production of SCFAs. In conclusion, *L. acidophilus* C4 can alleviate DSS-induced colitis by repairing mucosal barriers and maintaining intestinal microecological balance. The components of *L. acidophilus* C4 responsible for the alleviation of colitis are under investigation. Altogether, our findings demonstrated a great potential of *L. acidophilus* C4 for colitis treatment.

## Data availability statement

The data presented in this study are deposited in the NCBI repository, accession number ON171206. The 16S rRNA sequence of *L. acidophilus* C4 can also be found in the Supplementary material.

## Ethics statement

The animal study was reviewed and approved by the Ethics Committee of Chongqing Medical University (No. 2022178).

## Author contributions

QL and ZT designed the study, wrote the manuscript, and analyzed the data. QL, WJ, and LW performed the experiments. SY, SX, YN, KH, KC, and ZT reviewed and edited the manuscript. YZ, YG, and ZT funded the research. All authors contributed to the article and approved the submitted version.

## Footnotes

1
https://github.com/OpenGene/fastp

2
http://www.cbcb.umd.edu/flash
