# Supplementary material for: Alleviation of DSS-induced colitis in mice by a new-isolated Lactobacillus acidophilus C4
Source: Front Microbiol. 2023 Apr 20;14:1137701. doi: 10.3389/fmicb.2023.1137701 (PMC10157218; doi:10.3389/fmicb.2023.1137701)
Supplement: Supplementary file 1 [file Image_1.PDF]

Supplementary Figure S1: Analysis of intestinal microbiota

A

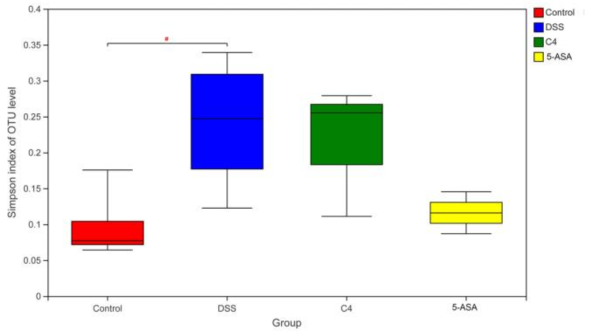

B

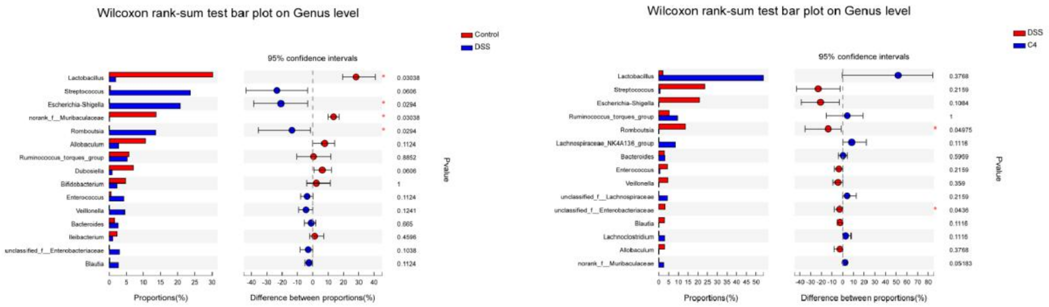

Effect of the C4 on Alpha diversity of intestinal microbiota at the OTU level. Data are subjected to T-test statistics and presented as mean ± standard deviation (n=4). (A) Simpson index of OTU level. (B) Rank-sum t-test for the composition of the gut microbiota at the genus level. *P < 0.05, **P < 0.01,***P < 0.001,****P < 0.0001 vs. DSS group; #P < 0.05, ##P < 0.01 vs. Normal group. (The experiment was repeated once).
