# Supplementary material for: Alleviation of DSS-induced colitis in mice by a new-isolated Lactobacillus acidophilus C4
Source: Front Microbiol. 2023 Apr 20;14:1137701. doi: 10.3389/fmicb.2023.1137701 (PMC10157218; doi:10.3389/fmicb.2023.1137701)
Supplement: Supplementary file 2 [file Table_1.PDF]

Supplementary Table S1 Determination of the intestinal colonization ability of *L. acidophilus* C4

Acid resistance and bile salt resistance of C4

Strain	Acid-resistant survival rate	Bile-tolerant salt survival rate
C4	53.57%	80%

Surface hydrophobicity rate and surface self-aggregation rate of *L. Acidophilus* C4

Experiment	Time（h）	OD_{A0}	OD_{A1}	Surface hydrophobicity	Surface self-cohesion
Surface hydrophobicity	0.25h	0.573	0.045	92.08%	—
Surface self-cohesion	0h	1.476	1.476	—	0
	1h	1.476	1.449	—	1.81%
	3h	1.476	1.409	—	4.52%
	5h	1.476	0.971	—	34.21%
	6h	1.476	1.03	—	30.22%
	24h	1.476	0.223	—	84.94%
