# Supplementary material for: Alleviation of DSS-induced colitis in mice by a new-isolated Lactobacillus acidophilus C4
Source: Front Microbiol. 2023 Apr 20;14:1137701. doi: 10.3389/fmicb.2023.1137701 (PMC10157218; doi:10.3389/fmicb.2023.1137701)
Supplement: Supplementary file 4 [file Table_3.docx]

| CTGAGTTTGATCCTGGCTCAGGACGAACGCTGGCGGCGTGCCTAATACATGCAAGTCGAGCGAGCTGAACCAACAGATTCACTTCGGTGATGACGTTGGGAACGCGAGCGGCGGATGGGTGAGTAACACGTGGGGAACCTGCCCCATAGTCTGGGATACCACTTGGAAACAGGTGCTAATACCGGATAAGAAAGCAGATCGCATGATCAGCTTATAAAAGGCGGCGTAAGCTGTCGCTATGGGATGGCCCCGCGGTGCATTAGCTAGTTGGTAGGGTAACGGCCTACCAAGGCAATGATGCATAGCCGAGTTGAGAGACTGATCGGCCACATTGGGACTGAGACACGGCCCAAACTCCTACGGGAGGCAGCAGTAGGGAATCTTCCACAATGGACGAAAGTCTAATGGAGCAACGCCGCGTGAGTGAAGAAGGTTTTCGGATCGTAAAGCTCTGTTGTTGGTGAAGAAGGATAGAGGTAGTAACTGGCCTTTATTTGACGGTAATCAACCAGAAAGTCACGGCTAACTACGTGCCAGCAGCCGCGGTAATACGTAGGTGGCAAGCGTTGTCCGGATTTATTGGGCGTAAAGCGAGCGCAGGCGGAAGAATAAGTCTGATGTGAAAGCCCTCGGCTTAACCGAGGAACTGCATCGGAAACTGTTTTTCTTGAGTGCAGAAGAGGAGAGTGGAACTCCATGTGTAGCGGTGGAATGCGTAGATATATGGAAGAACACCAGTGGCGAAGGTGGCTCTCTGGTCTGCAACTGACGCTGAGGCTCGAAAGCATGGGTAGCGAACAGGATTAGATACCCTGGTAGTCCATGCCGTAAACGATGAGTGCTAAGTGTTGGGAGGTTTCCGCCTCTCAGTGCTGCAGCTAACGCATTAAGCACTCCGCCTGGGGAGTACGACCGCAAGGTTGAAACTCAAAGGAATTGACGGGGGCCCGCACAAGCGGTGGAGCATGTGGTTTAATTCGAAGCAACGCGAAGAACCTTACCAGGTCTTGACATCTAGTGCAATCCGTAGAGATACGGAGTTCCCTTCGGGGACACTAAGACAGGTGGTGCATGGCTGTCGTCAGCTCGTGTCGTGAGATGTTGGGTTAAGTCCCGCAACGAGCGCAACCCTTGTCATTAGTTGCCAGCATTAAGTTGGGCACTCTAATGAGACTGCCGGTGACAAACCGGAGGAAGGTGGGGATGACGTCAAGTCATCATGCCCCTTATGACCTGGGCTACACACGTGCTACAATGGACAGTACAACGAGGAGCAAGCCTGCGAAGGCAAGCGAATCTCTTAAAGCTGTTCTCAGTTCGGACTGCAGTCTGCAACTCGACTGCACGAAGCTGGAATCGCTAGTAATCGCGGATCAGCACGCCGCGGTGAATACGTTCCCGGGCCTTGTACACACCGCCCGTCACACCATGGGAGTCTGCAATGCCCAAAGCCGGTGGCCTAACCTTCGGGAAGGAGCCGTCTAAGGCAGGGCAGATGACTGGGGTGAAGTCGTAACAAGGTAACCGTA |
| --- |

**Supplementary Table S3** 16S rRNA sequence of *Lactobacillus acidophilus* C4.
